# Josef Albers’ *Structural Constellations:* Investigating Formulations of Laminated Plastics Through Correlating the Industrial Literature with Scientific Analysis

**DOI:** 10.3390/polym17050681

**Published:** 2025-03-04

**Authors:** Maria Kokkori, Hortense de La Codre, Madeline C. Meier

**Affiliations:** Center for Scientific Studies in the Arts, Northwestern University/The Art Institute of Chicago, 2145 Sheridan Rd, Evanston, IL 60208, USA; hortense.delacodre@northwestern.edu (H.d.L.C.); madeline.meier@northwestern.edu (M.C.M.)

**Keywords:** Josef Albers, *Structural Constellations*, FTIR spectroscopy, laminated plastics

## Abstract

Josef Albers’ *Structural Constellations* series, created between 1948 and 1966, represents a pioneering exploration of plastic laminates as an artistic medium. Leveraging the unique properties of these materials, including their smooth surfaces, vibrant coloration, and precision in router engraving, Albers created machine-engraved works featuring intricate geometric compositions. This study combines archival research with scientific analysis to examine over fifty artworks and archival samples from the Josef and Anni Albers Foundation (1948–1970). Fourier-transform infrared (FTIR) spectroscopy and digital microscopy were employed to identify polymer types and analyze surface morphologies. Chemometric methods were applied to process the substantial dataset, offering key insights into Albers’ evolving material choices and their impact on the visual and structural properties of his works.

## 1. Introduction

In 1948, the Bauhaus artist Josef Albers began a series of machine-engraved drawings on plastic laminates, which he entitled *Structural Constellations*. From 1948 to 1966, Albers leveraged the inherent qualities of laminates, including their smooth surfaces, dense and vibrant coloration, and the precision of router engraving. This technique enabled the creation of lines of varying thickness, exposing the finely textured, white, or off-white substrate beneath [1,2].

The *Structural Constellations* series highlights Albers’s fascination with the fallibility and flexibility of visual perception through stark, linear compositions. The initial works, titled *Transformations of a Scheme* (1948–1952), convey a sense of movement and unresolved geometries by rotating and overlapping squares. Albers amplified this effect by selectively emphasizing specific intersection lines while erasing others, resulting in dynamic and ambiguous visual interactions. Between 1953 and 1966, the series—now named *Structural Constellations* to include all its iterations—expanded its focus on fluctuating, dynamic compositions. Albers incorporated interlocking forms, broadening his visual vocabulary. These intricate arrangements emerged through an iterative process of sketching on graph paper and architect’s trace. From hundreds of variations, he carefully selected those that struck a refined balance between simplicity and ambiguity, later transforming them into precise laminate engravings. In *Structural Constellations*, Albers predominantly used black-over-white laminates but did make four engravings using a matte-surfaced gray laminate. Originally developed for applications such as electrical insulation, interior cladding, signage, and machine operation panels, these laminates provided a novel medium for artistic exploration. In his limited notes on *Structural Constellations,* Albers referenced materials using trade names like Formica, Vinylite, and Resopal. However, these brand names do not consistently align with specific chemical compositions. For instance, phenol-formaldehyde resin was the first commercially available binding medium for laminated plastics, followed by urea-formaldehyde resin, which produced lighter-colored laminates. By 1939, melamine-formaldehyde resin emerged as a binding and impregnating medium for laminated plastics, further expanding their applications [3,4]. These innovative plastic materials, among others, opened new avenues for experimentation and creativity among modernist artists.

This research integrates archival investigations into industrial laminate plastic formulations, as documented in the German and U.S. industrial literature, with scientific analysis of over fifty artworks by Josef Albers and reference archival samples from the Josef and Anni Albers Foundation, dating from 1948 to 1970 (some undated). Digital microscopy was employed to visualize the surfaces of the artworks while Fourier-transform infrared (FTIR) spectroscopy in reflectance mode was used to identify the polymer types of the laminates. FTIR spectroscopy is the preferred method to characterize this type of materials as demonstrated in the literature within various museum collections [5,6,7,8,9]. Its non-contact and portable nature make it particularly well-suited for the analysis of artworks, allowing in situ investigations. Given the substantial dataset, a chemometric method, namely Principal Component Analysis (PCA), was applied for data processing. This method has been widely used in the cultural heritage field to process FTIR spectra [10,11,12] and visualize trends in a large dataset. This approach yielded valuable insights into changes in Albers’ material choices and the potential impact of individual laminates on the visual characteristics and long-term stability of his artworks.

This project was carried out in collaboration with the Josef and Anni Albers Foundation which has extensively researched and documented the contextual history, compositional evolution, production methods, and current whereabouts of Albers’s paintings [13,14,15,16,17,18]. Despite the substantial scholarship on Albers’s life and work, the laminated plastics he employed have received surprisingly little attention. These materials, vital to his engravings, remain largely unexplored in terms of their composition and their chemical and visual properties. This study aims to provide new insights into Albers’s innovative use of industrial materials, which were not only integral to his aesthetic but emblematic of his engagement with modernity and experimental practices. Thus, the study is particularly noteworthy as it represents the first comprehensive analysis of Josef Albers’ *Structural Constellations* on such a large scale. By examining a substantial collection of his works simultaneously, the research provides a unique opportunity to explore Albers’ artistic approaches, thematic evolution, and material variations across his oeuvre. The scope of this investigation not only deepens our understanding of Albers’ creative practice but also establishes a foundational reference for future research into his legacy. Employing this project as a framework, this paper aims to elucidate the technological aspects of laminated plastics, their visual exploitation by Albers, and the influence of material composition on the preservation and longevity of artworks created with these media.

## 2. Material and Methods

### 2.1. Structural Constellations

**Figure 1:**
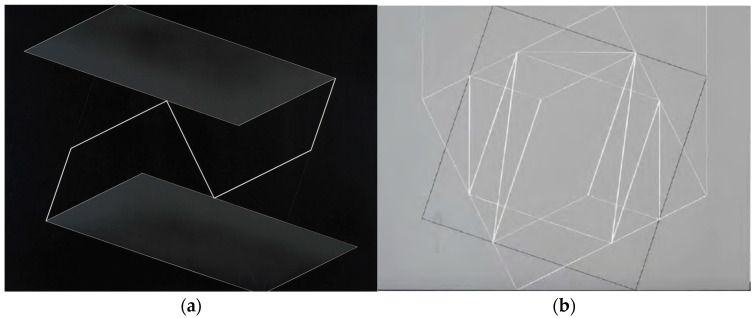
(**a**) Josef Albers, *Structural Constellation*, c. 1954. Machine-engraved and sandblasted black laminated plastic mounted on wood, 45.4 × 57.1 × 2 cm, (17 7/8 × 22 ½ × 13/16 in), #1976.8.1708; © Collection Josef and Anni Albers Foundation. (**b**) Josef Albers, *Transformation of a Scheme No. 5*, c. 1949. Machine-engraved gray plastic laminate mounted on wood, 43.1 × 57.1 × 2 cm, (17 22 ½ × 13/16 in.), #1976.8.1723; © Collection Josef and Anni Albers Foundation.

Josef Albers’ *Structural Constellations* series comprises over 200 artworks crafted from laminated plastics with black, gray, transparent, or wood-like appearances. These works, produced in a variety of sizes, are distinguished by the precise arrangement of simple geometric shapes that serve as the foundational elements for intricate compositions. Early engravings from the late 1940s through the early 1950s were executed in machine shops in New York, New Jersey, and Cincinnati. Later engravings of the 1960s are presumed to have been made at shops in New York and New Haven. The larger, unique engravings were customarily mounted onto thick pieces of black-painted plywood with an inversely beveled edge. A few works were mounted onto thinner pieces of plywood and surrounded with steel and aluminum frames. The complexity of these compositions is achieved through machine-engraved lines of varying thickness and depth, forming geometric patterns, as illustrated in the representative example shown in Figure 1.

In this study, a total of 53 artworks were analyzed. Of these, fifty are housed at the Josef and Anni Albers Foundation, and three are part of the collection at the Yale University Art Gallery. Table 1 provides a detailed summary of the artworks studied, including their reference numbers, dates, and the color of the laminated plastic used.

### 2.2. Archival Research

Archival research was carried out at the Smithsonian National Museum of American History Archives Center. Fifteen boxes from the Formica Collection, 1913–2003 were investigated [19]. Archival materials also include the entire eight boxes of the J. Harry DuBois Collection on the History of Plastics [20].

### 2.3. Data Acquisition and Processing

#### 2.3.1. Digital Microscope Observation

Microscope observations were performed using a digital microscope USB long working distance ©Edge Dino-Lite AM7915MZT(Dino-Lite, Waltham, MA USA) with a magnification from ×10 to ×220 and a 2592 × 1944 pixels resolution. ©Dinocapture software 2.0 (version 1.5.48) was used to control the microscope and acquire micrographs. The areas selected for analysis by microscopy were chosen systematically to identify and visualize representative geometric features and surface morphologies for each work. Multiple magnifications were utilized to better capture features of varying sizes.

#### 2.3.2. Fourier-Transform Infra-Red (FTIR) Spectroscopy

A Bruker Alpha spectrometer, equipped with a specular reflectance head was used. Data were collected in reflectance mode between 360 cm^−1^ and 7000 cm^−1^ at 4 cm^−1^ resolution and 256 scans per spectrum. Three different spectra locations were recorded for each of the artworks on the smooth surfaces. Selections of analyzed areas were informed by the previous micrographic analysis and selected in a systematic way. Recording and curation of spectra were performed with ©OPUS_7.0.129 Software and identification was completed through comparison with the IRUG library spectra.

#### 2.3.3. Principal Components Analysis (PCA)

Principal components analysis was applied to the FTIR dataset. To use this method, the three spectra recorded on each artwork were averaged and underwent two pretreatment steps performed with ©OMNIC 9.1.27 software: a Kramers-Kronig transformation to convert the spectra into absorption data [5] followed by a Savitzky-Golay derivative (derivative order 2, with 13 smoothing points and a polynomial order of 2). Those choices have been motivated by the previous literature discussing the most appropriate pretreatments in the case of FTIR spectra applied to PCA [12]. PCA was then performed on this dataset using Galaxy Chemflow 20.05 software, with a maximum number of PCs set at 10.

## 3. Results

### 3.1. Archival Findings

The archival materials from the Formica Collection included a broad range of items, such as patents, advertisements, product descriptions, brochures, and business records documenting the locations of the Formica Company’s distribution and sales centers. Similarly, the J. Harry Dubois collection contained relevant, though less extensive, materials on plastic manufacturers, companies, and innovations in plastics, dating from the late 1800s to the mid-1900s.

**Figure 2:**
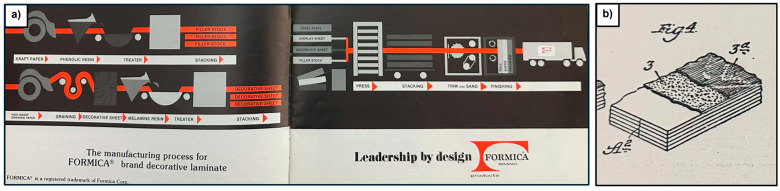
Archival materials from the Formica Collection. (**a**) Undated brochure from the Formica Company providing an overview of the laminate manufacturing process. (**b**) Figure from US patent No. 1,997,358 detailing the layers in the improved laminate formulations.

Figure 2a illustrates an example of the archival materials examined: a production brochure from the Formica Corporation. This brochure provides a simplified overview of the laminate manufacturing process, highlighting key material components such as Kraft paper, phenolic resin, and melamine resin. It also outlines the layering and processing steps involved in producing laminate materials.

In addition to advertisements, business logistics, and correspondence related to the Formica Corporation, both collections contained patents that provided chemical insights and documented advancements in laminate materials, including urea-formaldehyde and phenolic resin-based compounds. Several U.S. patents (Nos. 1,863,239, 1,904,718, and 1,956,314) detailed methods for enhancing the surface designs of laminate materials. These patents specifically aimed to enhance the depth and contrast of surface design sheets, improving their ability to replicate wood grain and texture by incorporating broader color ranges and greater variations across the laminate surface.

Two notable patents from the Formica Collection, U.S. Patent Nos. 1,997,358 (1935) and 2,038,345 (1936), discussed improvements in urea-formaldehyde coatings aimed at mitigating a phenomenon known as “crazing”—the formation of fine cracks that detract from a pristine surface appearance. The patents proposed a reformulated varnish mixture comprising urea-formaldehyde resin, titanium oxide white pigments, and finely ground paper fiber to minimize this issue. Figure 2b illustrates a diagram from one of these patents, depicting the layering of materials in laminate product formation. Layer A2 consists of “body sheets”, while layers 3 and 3a represent a “barrier sheet” and “surface sheet”, respectively. The barrier sheet prevents the darker color of the body sheets from affecting the visual appearance of the surface. Both the surface and barrier sheets incorporate the reformulated urea-formaldehyde resin, available in clear or dyed formulations, depending on the desired product appearance.

Additionally, U.S. Patent No. 1,551,428 from the Formica Collection describes the process and production of resin-containing fibrous mixtures. This patent outlines the composition and proportions of materials, including non-reactive phenol resin, commercial phenol, paper pulp, and hexamethylenetetramine (with formaldehyde or other aldehydes as alternatives). The full texts of these patents, including the figures and technical details, are provided in the Supplemental Information.

### 3.2. Visual and Microscopic Investigations of Works

#### 3.2.1. Topography and Engraved Lines

**Figure 3:**
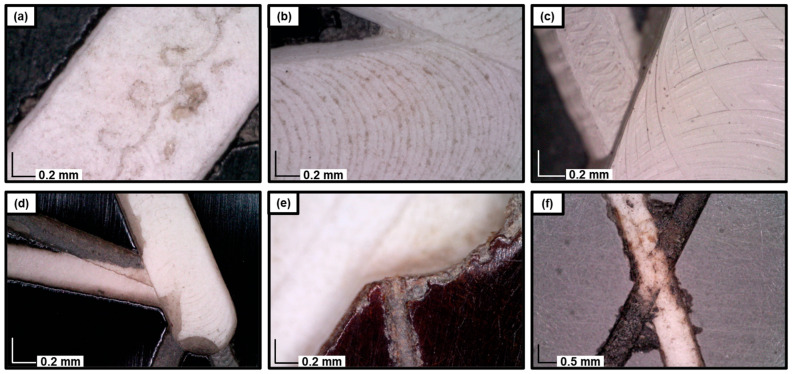
Micrographs (×190) of (**a**) #1976.8.1750; (**b**) #1976.8.1717.; (**c**) #1976.8.1901; (**d**) #1976.8.1714; (**e**) #1976.8.1725; (**f**) #1976.8.1723 (×60).

Optical microscopy of the engravings revealed several distinct features. The micrographs shown in Figure 3a–c illustrate unique markings indicative of routers of varying types and sizes used to fabricate the geometric forms in the artworks. Differences in the router tools are evident in the contrast between the micrograph in Figure 3a and those in Figure 3b,c, which show distinct patterns: the former displays the repetition of multiple small loops, whereas the latter exhibits larger circular impressions. Although the size scales of the micrographs in panels b and c are similar, noticeable differences in the circular patterns are apparent. Specifically, the stacking of multiple layers of material in the artworks likely influences the transmission or constraint of circular movements between layers, depending on the material type. This difference is also evident in the white layer’s appearance, which shows greater reflectivity in panel c compared to the more textured surface observed in panel b. These differences suggest that the observed movement patterns are primarily influenced by variations in material properties rather than the choice of tools or engraving methods.

Micrographs in Figure 3d,e provide insights into the engraving sequence, revealing two types of engraved colors: white and off-black. Figure 3d demonstrates that thinner lines were engraved before thicker ones, as evidenced by the superposition of a thick white engraving over two thinner engravings at the intersection of three lines. Figure 3e highlights differences in engraving depth, allowing for the observation of the layered sheet structure along the edges of the engravings, as illustrated in Figure 2b. The variation in engraving depth corresponds to differences in color, with the deeper white engravings appearing more prominent than the shallower gray ones.

Finally, the micrograph in Figure 3f captures a unique feature observed exclusively in the artwork #1976.8.1723 (Figure 1b). Specifically, two distinct markings are visible: a white engraving consistent with those found in other works and a grayish-black marking that appears to have been infilled or tinted with ink. Additional micrographs (not shown) revealed the diffusion of ink into the material, further distinguishing this engraving from others in the series.

#### 3.2.2. Surface Morphology and Patterning

**Figure 4:**
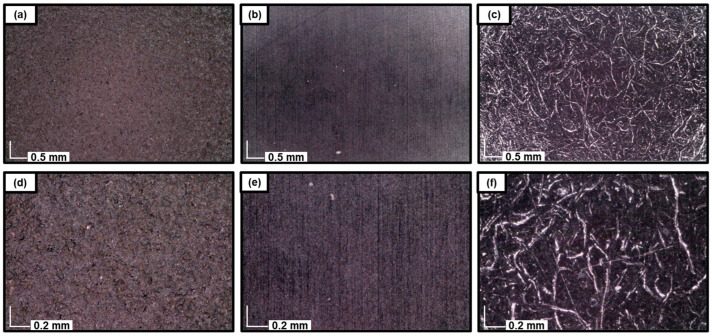
Micrographs of the black laminated surfaces at a magnification of ×60 (**a**–**c**) and ×190 (**d**–**f**) from #1976.8.1704, #1976.8.1760, and #1976.8.1903, respectively.

Microscopic analyses of the black laminate surfaces (Figure 4) provided detailed insights into their surface morphologies. Each panel in Figure 4 represents one of the three primary surface appearances identified in this study. Figure 4a,d show regions of a Structural Constellation artwork that exhibit characteristics of sandblasting, as evidenced by the surface roughness and granular texture. This sandblasted appearance is distinct and suggests intentional surface modification. The most commonly observed surface morphology among the 53 examined works, depicted in Figure 4b,e, displays a smooth, continuous texture consistent with standard descriptions of laminate materials. This smooth appearance aligns with the inherent properties of laminated surfaces and represents the typical untreated morphology. However, not all artworks exhibited this smoother surface texture. Figure 4c,f illustrate surface deformations characterized by fibril-like and void features. These features resemble stress-induced cracking or crazing, phenomena known to occur in certain polymeric materials [21,22].

Table 1 categorizes the examined works based on the presence of sandblasting features (Figure 4a,d) or crazing patterns (Figure 4c,f). The observed surface conditions appear to arise from a combination of factors, reflecting both intentional artistic interventions and the natural behavior of the material. For example, the sandblasted regions likely represent deliberate choices by Albers to achieve specific visual or textural effects. In contrast, the smooth surface morphology (Figure 4b,e) is intrinsic to the material and represents its original state. However, the crazing patterns (Figure 4c,f) are more likely attributable to degradation processes over time, highlighting the material’s vulnerability. These patterns, rather than being an artistic choice, likely reflect the natural aging and stress responses of the laminate material.

### 3.3. Material Identification

Fourier transform infra-red (FTIR) spectroscopy has been widely employed to characterize plastic materials since it allows rapid, noninvasive analysis. When portable instruments are used, it also enables in situ examination of objects in galleries or storage settings. This spectroscopic method reliably identifies the polymer components of plastics, with additional absorption peaks in the spectra often providing information about plasticizers or other additives present [6,23,24,25]. This approach provided valuable insights into the materials used by Albers while maintaining the integrity of the artworks.

In this study, 53 *Structural Constellations* were analyzed. Due to the substantial number of FTIR spectra requiring evaluation, statistical tools were employed to enhance efficiency and minimize the risk of misidentification. Principal component analysis was applied to the 53 r-FTIR spectra of the surfaces, enabling systematic interpretation and identification of spectral patterns.

**Figure 5:**
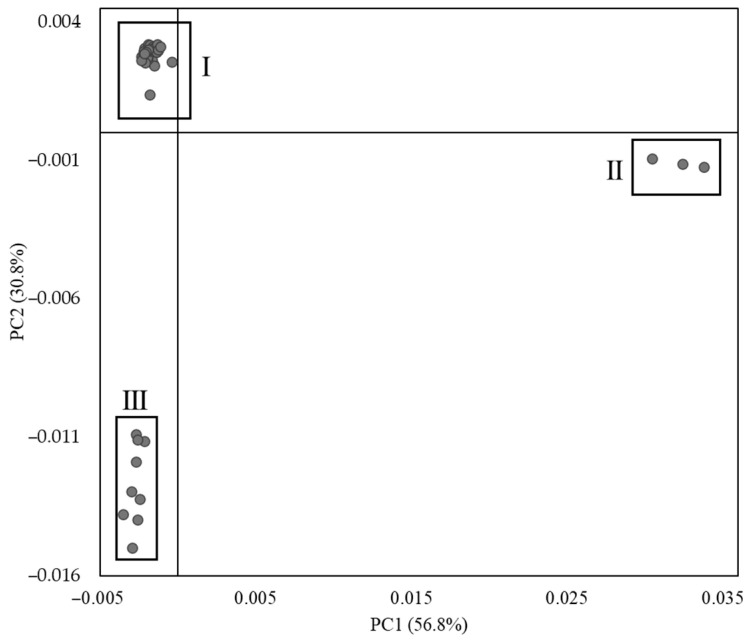
Factorial map (PC1 × PC2). The 53 averaged spectra have undergone a Kramers-Kronig transformation followed by a Savitzky-Golay derivative (derivative order 2, with 13 smoothing points and a polynomial order of 2). The composition of groups I, II and III is described in the main text Section 3.3.

The factorial map of principal components 1 and 2 (PC1 and PC2), presented in Figure 5, reveals three distinct sample groups (I, II and III), with a cumulative explained variance of 87.6%. Eigenvalues and explained variance % for the 10 PC as well as loadings for PC1 and PC2 can be found in the SI section. This high cumulative explained variance indicates that the PCA model effectively captures the primary structure of the data. The classification of each artwork within these groups is detailed in Table 1. Notably, not all *Structural Constellations* are composed of black laminates, which could have introduced variability in the PCA results. However, as shown in Table 1, samples within Groups I, II, and III are consistently clustered together despite differences in coloration. This finding suggests that coloration does not significantly influence the PCA calculations for PC1 and PC2. This may be attributed to the use of small amounts of organic colorants, which produce minimal or undetectable peaks in the FTIR spectra, or to the possibility that PC1 and PC2 are not the most sensitive components for capturing variations in color. Despite this, the clustering achieved using PC1 and PC2 effectively reflects the artworks’ primary material composition, underscoring the method’s robustness in distinguishing material groups. Therefore, PCA proved to be an effective tool for classifying a large dataset into distinct material groups, streamlining the identification process. By enabling the automatic clustering of materials, PCA eliminated the need to manually determine the composition of each of the 53 artworks individually. This data-driven approach not only significantly reduced analysis time but also minimized the risk of human error, enhancing the reliability and efficiency of the material identification process.

**Figure 6:**
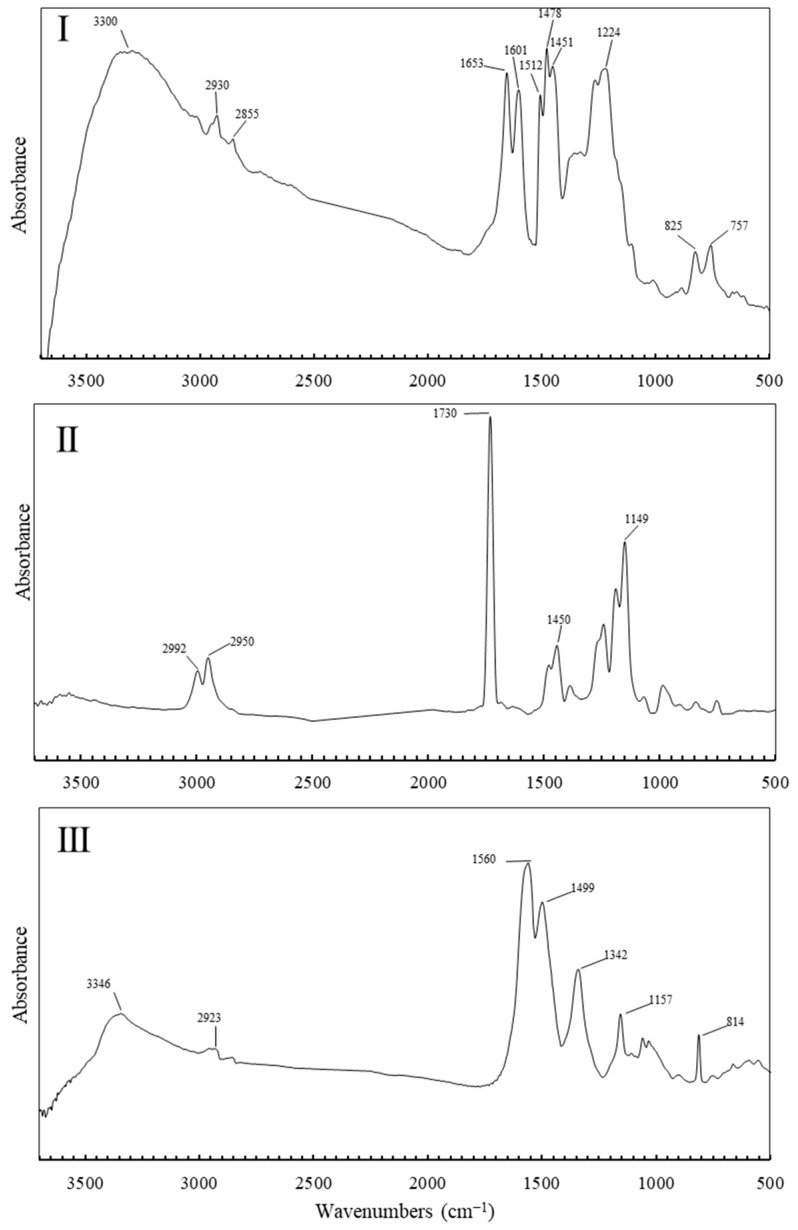
r-FTIR spectrum of sample: (**I**) #1976.8.1708; (**II**) #1976.8.1901; (**III**) #1976.8.1724. The spectra are shown in absorbance after a Kramers-Kronig transformation.

Figure 6 presents representative spectra for each group, enabling the identification of materials associated with each artwork group based on their spectral characteristics. The spectrum for Group I indicates the use of a urea-formaldehyde (UF) resin mixed with phenolic resin, as supported by the following peak assignments: a broad peak around 3300–3400 cm^−1^ indicating N–H stretching, characteristic of amine or amide groups in urea; the peak at 2930 cm^−1^ suggests C–H stretching from alkyl groups, associated with the formaldehyde component. The peak at 1653 cm^−1^, in the 1650–1700 cm^−1^ region corresponds to C=O stretching, typical of the carbonyl groups in urea, while the peak at 1601 cm^−1^ can be attributed to N–H bending vibrations, further confirming the presence of amide linkages in the resin. The peaks observed at 1512 cm^−1^, 1475 cm^−1^, and 1451 cm^−1^ in the spectrum are attributed to the C=C stretching vibrations of the aromatic rings (1512 cm^−1^) and the in-plane scissoring and asymmetric deformation modes of the methylene (–CH_2_–) bridges (1475 cm^−1^ and 1451 cm^−1^), respectively, which are characteristic of the chemical structure of phenolic resins. The band at 1219 cm^−1^ is indicative of C–N stretching vibrations, which are common in urea-formaldehyde resins. Finally, the peak at 825 cm^−1^ is associated with out-of-plane C–H bending vibrations, contributing to the overall characterization of the resin. Collectively, these peaks provide a fingerprint of the urea-formaldehyde resin’s structure likely mixed with phenolic resin. The spectrum of Group II suggests the use of poly(methyl methacrylate) (PMMA) [26,27,28]. The spectra contain two peaks at 2992 cm^−1^ and 2950 cm^−1^ attributed to the stretching vibrations of C–H bonds of aliphatic carbon for methyl groups (–CH_3_) and methylene groups (–CH_2_–), respectively. The sharp peak at 1730 cm^−1^ can be associated with the stretching of an ester carbonyl group (C=O). The signal at 1450 cm^−1^ indicates the bending vibrations of C–H bonds present whereas the peak at 1130 cm^−1^ in the presence of PMMA is typically attributed to the stretching vibrations of the C–O bond, indicative of the ester functional group present in the polymer structure. The third spectrum indicates the use of melamine-formaldehyde, as evidenced by several characteristic peaks. A broad peak between 3300 and 3500 cm^−1^ corresponds to N–H stretching vibrations, which are prominent due to the high nitrogen content of melamine. A peak at 2923 cm^−1^ is typically associated with the C–H stretching vibrations of aliphatic CH_2_ and CH_3_ groups, indicating the presence of methylene (–CH_2_–) and/or methyl (–CH_3_) groups within the polymer structure. In the 1550–1650 cm^−1^ range, peaks arise from C=N stretching and N–H bending, reflecting the presence of melamine’s triazine rings, each containing three C=N bonds within a six-membered ring structure. The peak at 1499 cm^−1^ is attributed to the C–N stretching vibration, indicating the presence of nitrogen-containing functional groups in the polymer structure. Additionally, the spectrum shows a peak at 1339 cm^−1^ and peaks between 1340 and 1450 cm^−1^ associated with C–N stretching, highlighting the nitrogen-rich bonds connecting the triazine rings, which are a core component of the resin’s network. The peak at 1157 cm^−1^ corresponds to C–O or C–O–C stretching vibrations, typical of ether or ester linkages. Finally, the distinct peak around 814 cm^−1^ corresponds to the out-of-plane bending of N–H or C–N bonds, a signature of the triazine ring system. Together, these spectral features confirm the presence of a highly cross-linked, nitrogen-rich network characteristic of melamine-formaldehyde [29].

## 4. Discussion and Conclusions

Microscopic observations revealed notable differences in both the choice of materials and the artist’s interventions on these materials. One key finding was the use of various engraving tools, with differences in tool size employed to create lines of varying thickness. Additionally, the engraving depth was deliberately altered to produce color variations.

The ability to manipulate engraving depth appears to have been particularly significant to the artist, as it allowed for the intentional modulation of visual perception and texture within the artworks. By controlling the depth of the engravings, the artist influenced the way light interacted with the surface, enhancing dimensionality and amplifying the visual impact of the compositions.

The significance of color in this context is further underscored by the intentional use of ink to enhance the engravings, as for example in the work #1976.8.1723. Although the engravings were carved to the same depth, the artist deliberately applied ink to introduce an additional layer of visual depth, enriching the viewer’s experience. The ink not only accentuates the engraved lines but also differentiates tonalities, thereby enhancing the contrast between the white and darker areas of the composition. This deliberate manipulation of both depth and color illustrates the artist’s intent to create a dynamic interplay between surface texture and perceived depth, adding complexity and nuance to the work. This intent is further demonstrated in the use of sandblasted areas, where the surface was purposefully altered to create additional contrast and texture. The sandblasting technique introduces another layer of variation in the material’s surface, contributing to the overall dimensionality of the artwork. By integrating these methods, the artist effectively enhanced the visual complexity of the compositions, highlighting a meticulous engagement with both material properties and artistic techniques. These interventions were carefully designed to influence the viewer’s perception, emphasizing the interplay between form, texture, and light to create a multifaceted visual experience.

The identification of Group I as a probable mixture of phenolic resin and urea–formaldehyde resin by FTIR is supported by archival evidence. Patent No. 1,551,428 describes the use of formaldehyde-based materials mixed with phenolic resin in the formulation of plastics after 1935, while Patent No. 2,038,345 details an improved urea-formaldehyde varnish resin designed to minimize “crazing”. Interestingly, all artworks exhibiting crazing morphology were composed of melamine–formaldehyde resin. This observation is particularly significant in light of archival information indicating that crazing was a well-documented issue in urea-based materials until the 1930s. During that period, advancements in resin formulations effectively addressed and mitigated the problem in urea-based materials. However, the same level of mitigation does not appear to have been achieved in melamine–formaldehyde resin, as evidenced by the persistent occurrence of crazing in the analyzed works. Notably, ongoing research continues to explore strategies to minimize these types of defects in melamine-formaldehyde materials [30].

These observations, combined with the dating of the works, provide valuable insights into Josef Albers’ technical and artistic choices throughout his career. The timeline reveals a consistent use of urea-formaldehyde resin from the creation of the first *Structural Constellations* in 1948 through to later works in 1966. This material appears to have been favored by the artist for its aesthetic and technical properties, serving as the foundation for the majority of his works during this period. In contrast, a smaller subset of works (nine) was created on melamine-formaldehyde supports (group III). Although four of these works are undated, the timeline indicates a sporadic use of this material, with dated examples appearing in 1948, 1949, and 1958. This limited use suggests that Albers did not regard melamine-formaldehyde with the same preference as urea-formaldehyde when producing his *Structural Constellations*. This may reflect the artist’s evolving material preferences as he experimented with different substrates, potentially seeking those that provided greater stability or more refined visual effects.

Finally, three works composed of PMMA (polymethyl methacrylate) have been identified, though they remain undated. These works represent a notable shift in material choice; however, the absence of precise dating makes it challenging to ascertain their exact place within Albers’ artistic progression. This timeline is of particular interest as it may provide insights into the artist’s evolving material preferences and decision-making processes. It is plausible that Albers, known for his meticulous attention to detail and sensitivity to the visual effects of his materials, may have been influenced by the macroscopic appearance of melamine-formaldehyde (MF), particularly its tendency to develop deformations or “crazing”. If these cracks or distress were perceived as detrimental to the aesthetic or structural integrity of his artworks, Albers may have consciously chosen to move away from MF in favor of urea-formaldehyde (UF) for the majority of his subsequent works. This shift could reflect a preference for a more stable, visually consistent surface, free from the undesirable effects of crazing that might have compromised the final presentation of the works. This transition in material use underscores Albers’ engagement with the properties of his chosen media and his evolving approach to texture and surface manipulation within his art. The artist’s awareness of the potential flaws in certain materials and his ability to adapt his choices accordingly highlight his deep understanding of the interplay between materiality, form, and perception in his work.

Among these three groups of materials, all include black works, though not exclusively. Each group also features works in other hues, such as transparent, gray, or wood-like appearances for Groups II, III, and I, respectively. The variety in color, combined with Albers’ experimentation with materials, highlights his thoughtful and deliberate approach to the interplay between materials and colors. Albers’ choice of materials was not merely functional; it was deeply connected to his exploration of how different surfaces and textures influenced the perception of color, depth, and light within his artworks. His reflective approach to materiality demonstrates a careful consideration of how form and color could be harmonized to enhance the overall visual experience. The deliberate integration of material properties with artistic intent underscores Albers’ innovative approach to texture, surface, and their role in shaping visual perception.

This work provided a unique opportunity to investigate Josef Albers *Structural Constellations* series from both historical and scientific perspectives. Digital microscopy and FTIR analysis allowed for visualization and material identification, respectively. These technical methods, further enhanced by the use of chemometrics, when combined with archival materials provided insight into Albers’ methodical process of material selection and his overall approach to materiality.

## Figures and Tables

**Table 1:** The identification number, date, color of laminated plastics, and surface topography and morphology, as determined by digital microscopy of the artworks.

ID Number	Date	Color	Size (cm)	Sandblasted Areas	Crazing	Group
1976.8.1726	1948	Gray	45.7 × 66.0	NO	*	III
1976.8.1761	1948	Black	33.8 × 62.3	NO	NO	I
1976.8.1913	1948	Black	24.1 × 48.2	NO	NO	I
1976.8.1723	1949	Gray	43.1 × 57.1	NO	YES	III
1976.8.1724	1949	Gray	43.1 × 57.1	NO	YES	III
1976.8.1892	1949	Gray	43.1 × 57.1	NO	YES	III
1976.8.1887	1949–1951	Black	43.1 × 57.1	NO	NO	I
1976.8.1704	1950–1951	Black	43.1 × 56.5	YES	NO	I
1976.8.1714	1950	Black	43.1 × 57.1	NO	NO	I
1976.8.1725	1950	Wood	43.1 × 57.1	NO	NO	I
1976.8.1885	1950	Black	43.1 × 57.1	YES	NO	I
1976.8.1891	1951	Black	43.1 × 57.1	YES	NO	I
1976.8.1750	1952	Black	43.1 × 57.1	YES	NO	I
1976.8.1756	1952	Black	43.2 × 57.1	YES	NO	I
1976.8.1763	1952	Black	43.1 × 57.1	YES	NO	I
1977.160.63	1952	Black	43.1 × 57.1	NO	NO	I
2016.8.1	1953	Black	43.1 × 57.1	NO	*	I
1976.8.1708	1954	Black	45.4 × 57.1	YES	NO	I
1976.8.1817	1954	Black	43.1 × 57.1	YES	NO	I
1976.8.1860	1954	Black	43.3 × 57.3	YES	NO	I
1976.8.1886	1954	Black	15.8 × 20.9	NO	NO	I
1977.160.64	1954	Black	15.8 × 20.9	NO	NO	I
1976.8.1904	1958	Black	16.9 × 22.8	NO	NO	I
1976.8.1908	1958	Black	49.5 × 66.0	NO	YES	III
1976.8.1905	1959	Black	50.8 × 66.6	NO	NO	I
1976.8.1893	1962	Black	49.5 × 65.4	NO	NO	I
1976.8.1703	1964	Black	12.7 × 34.9	NO	NO	I
2002.145.2	1964	Black	12.7 × 34.9	NO	NO	I
1976.8.1896	1966	Black	12.7 × 34.9	NO	NO	I
1976.8.1896a	1966	Black	12.7 × 34.9	NO	NO	I
1976.8.1897	1966	Black	43.1 × 57.1	NO	NO	I
1976.8.1897a	1966	Black	43.1 × 57.1	YES	NO	I
1976.8.1701	n.d.	Black	43.1 × 56.8	YES	NO	I
1976.8.1716	n.d.	Black	43.1 × 57.1	YES	NO	I
1976.8.1717	n.d.	Black	43.1 × 57.1	NO	NO	I
1976.8.1758	n.d.	Black	43.8 × 57.1	NO	NO	I
1976.8.1760	n.d.	Black	43.1 × 57.1	NO	NO	I
1976.8.1762	n.d.	Black	43.1 × 57.1	NO	NO	I
1976.8.1764	n.d.	Black	43.1 × 57.1	NO	NO	I
1976.8.1865	n.d.	Black	43.1 × 57.1	NO	NO	I
1976.8.1898	n.d.	Black	20.3 × 27.9	NO	NO	I
1976.8.1899	n.d.	Black	6.6 × 8.2	NO	YES	III
1976.8.1900	n.d.	Black	7.9 × 10.4	NO	YES	III
1976.8.1901	n.d.	Black	8.5 × 10.4	NO	NO	II
1976.8.1902	n.d.	Black	16.8 × 22.5	NO	NO	I
1976.8.1903	n.d.	Black	16.5 × 22.8	NO	YES	III
1976.8.1906	n.d.	Black	12.5 × 17.1	NO	YES	III
1976.8.1907	n.d.	Black	13.9 × 16.8	NO	NO	I
1976.8.1910	n.d.	Transparent	12.7 × 16.8	NO	NO	II
1976.8.1911	n.d.	Transparent	20.9 × 24.1	NO	NO	II
1976.8.1909	n.d. or 1964	Black	17.1 × 23.1	NO	NO	I
1976.8.1759	n.d. or 1965	Black	16.5 × 22.8	NO	NO	I
1976.8.1721	n.d.	Black	43.1 × 57.1	NO	NO	I

* Presence of crazing phenomena was hard to determine with confidence because of the work’s state of preservation. n.d. stands for not determined. The date of this specific artwork could not be determined by the Josef and Anni Albers Foundation.

## Data Availability

The data supporting the findings of this study are available within the article and its Supplementary Materials.

## Supplementary Materials

The following supporting information can be downloaded at: https://www.mdpi.com/article/10.3390/polym17050681/s1, Figure S1: Patent No. 1,997,358; Figure S2: Patent No. 2,038,345; Figure S3: Patent No. 1,551,428; Figure S4: Plot of loading for Principal Component 1; Figure S5: Plot of loading for Principal Component 2; Table S1: Explained variance percentage and eigenvalues for all 10 principal components.
