# Supplementary material for: Josef Albers’ Structural Constellations: Investigating Formulations of Laminated Plastics Through Correlating the Industrial Literature with Scientific Analysis
Source: Polymers (Basel). 2025 Mar 4;17(5):681. doi: 10.3390/polym17050681 (PMC11902680; doi:10.3390/polym17050681)
Supplement: Supplementary file 1 [file polymers-17-00681-s001.zip › polymers-3437036-supplementary.pdf]

Josef Albers' *Structural Constellations:* Investigating formulations of laminated plastics through the correlation of industrial literature with scientific analysis

Supplemental Information

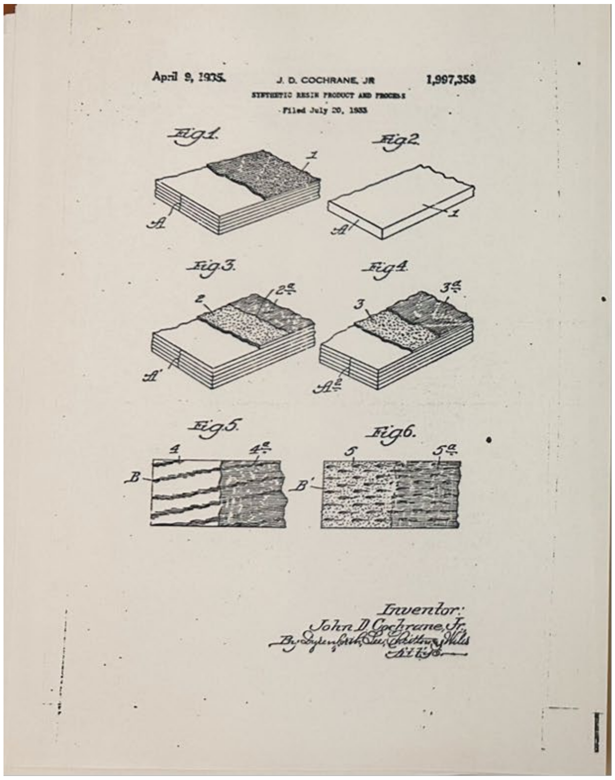

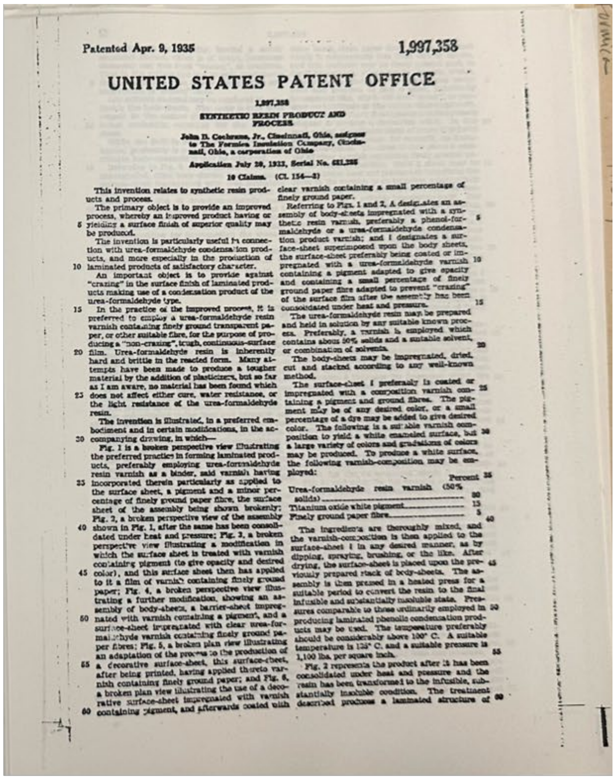

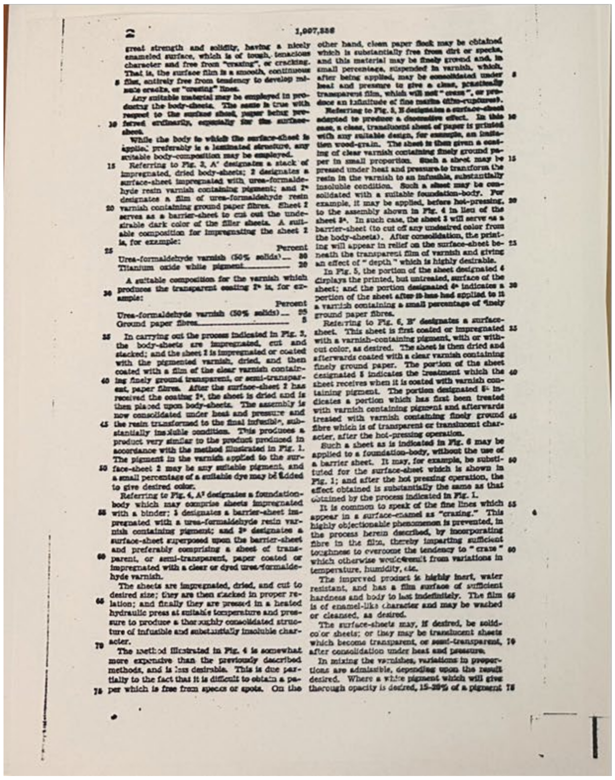

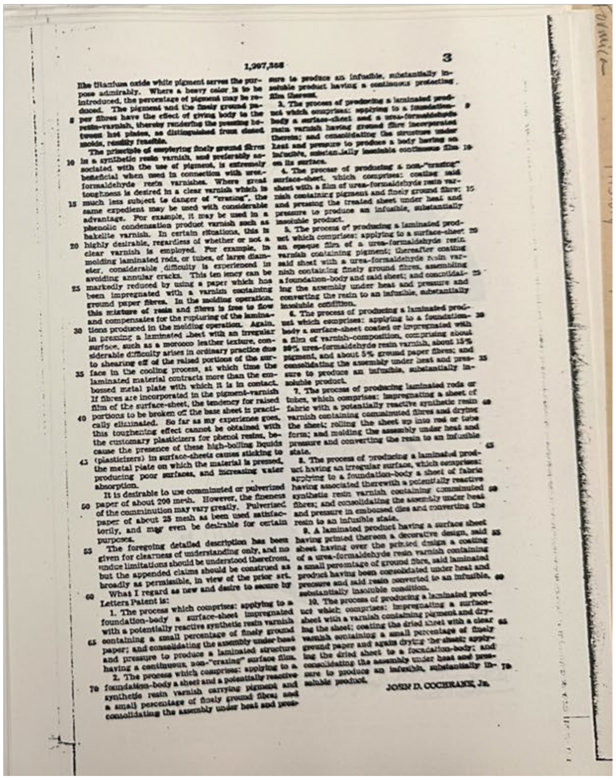

Figure S1: Patent No. 1,997,358

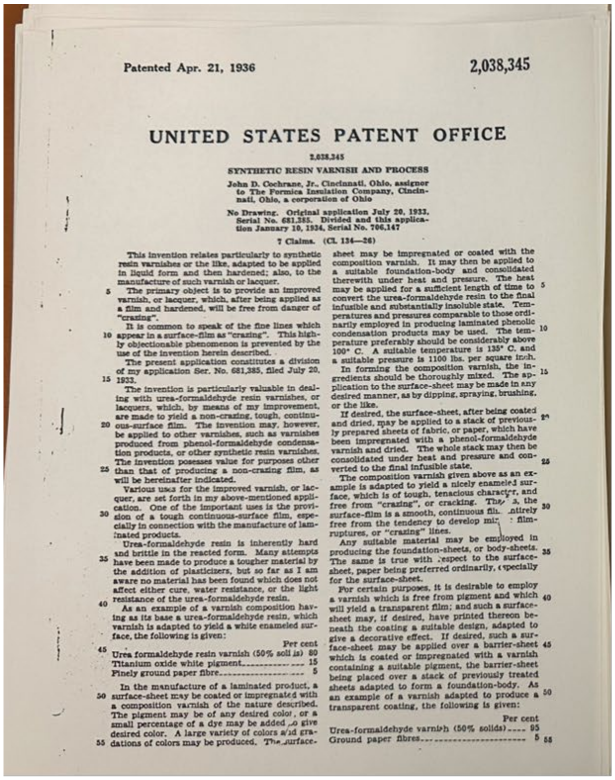

Patented Apr. 21, 1936

2,038,345

UNITED STATES PATENT OFFICE

2,038,345

SYNTHETIC RESIN VARNISH AND PROCESS

John D. Cochrane, Jr., Cincinnati, Ohio, assignor to The Formica Insulation Company, Cincinnati, Ohio, a corporation of Ohio

No Drawing. Original application July 20, 1933, Serial No. 681,385. Divided and this application January 10, 1934, Serial No. 706,147

7 Claims. (Cl. 134—26)

This invention relates particularly to synthetic resin varnishes or the like, adapted to be applied in liquid form and then hardened; also, to the manufacture of such varnish or lacquer.

The primary object is to provide an improved varnish, or lacquer, which, after being applied as a film and hardened, will be free from danger of "crazing".

It is common to speak of the fine lines which appear in a surface-film as "crazing". This highly objectionable phenomenon is prevented by the use of the invention herein described.

The present application constitutes a division of my application Ser. No. 681,385, filed July 20, 1933.

The invention is particularly valuable in dealing with urea-formaldehyde resin varnishes, or lacquers, which, by means of my improvement, are made to yield a non-crazing, tough, continuous-surface film. The invention may, however, be applied to other varnishes, such as varnishes produced from phenol-formaldehyde condensation products, or other synthetic resin varnishes. The invention posesses value for purposes other than that of producing a non-crazing film, as will be hereinafter indicated.

Various uses for the improved varnish, or lacquer, are set forth in my above-mentioned application. One of the important uses is the provision of a tough continuous-surface film, especially in connection with the manufacture of laminated products.

Urea-formaldehyde resin is inherently hard and brittle in the reacted form. Many attempts have been made to produce a tougher material by the addition of plasticizers, but so far as I am aware no material has been found which does not affect either cure, water resistance, or the light resistance of the urea-formaldehyde resin.

As an example of a varnish composition having as its base a urea-formaldehyde resin, which varnish is adapted to yield a white enameled surface, the following is given:

	Per cent
Urea formaldehyde resin varnish (50% solids)	80
Titanium oxide white pigment	15
Finely ground paper fibre	5

In the manufacture of a laminated product, a surface-sheet may be coated or impregnated with a composition varnish of the nature described. The pigment may be of any desired color, or a small percentage of a dye may be added to give desired color. A large variety of colors and gradations of colors may be produced. The surface-sheet may be impregnated or coated with the composition varnish. It may then be applied to a suitable foundation-body and consolidated therewith under heat and pressure. The heat may be applied for a sufficient length of time to convert the urea-formaldehyde resin to the final infusible and substantially insoluble state. Temperatures and pressures comparable to those ordinarily employed in producing laminated phenolic condensation products may be used. The temperature preferably should be considerably above 100° C. A suitable temperature is 135° C. and a suitable pressure is 1100 lbs. per square inch.

In forming the composition varnish, the ingredients should be thoroughly mixed. The application to the surface-sheet may be made in any desired manner, as by dipping, spraying, brushing, or the like.

If desired, the surface-sheet, after being coated and dried, may be applied to a stack of previously prepared sheets of fabric, or paper, which have been impregnated with a phenol-formaldehyde varnish and dried. The whole stack may then be consolidated under heat and pressure and converted to the final infusible state.

The composition varnish given above as an example is adapted to yield a nicely enameled surface, which is of tough, tenacious character, and free from "crazing", or cracking. Th[illegible], the surface-film is a smooth, continuous fil[illegible] entirely free from the tendency to develop mi[illegible] film-ruptures, or "crazing" lines.

Any suitable material may be employed in producing the foundation-sheets, or body-sheets. The same is true with respect to the surface-sheet, paper being preferred ordinarily, especially for the surface-sheet.

For certain purposes, it is desirable to employ a varnish which is free from pigment and which will yield a transparent film; and such a surface-sheet may, if desired, have printed thereon beneath the coating a suitable design, adapted to give a decorative effect. If desired, such a surface-sheet may be applied over a barrier-sheet which is coated or impregnated with a varnish containing a suitable pigment, the barrier-sheet being placed over a stack of previously treated sheets adapted to form a foundation-body. As an example of a varnish adapted to produce a transparent coating, the following is given:

	Per cent
Urea-formaldehyde varnish (50% solids)	95
Ground paper fibres	5

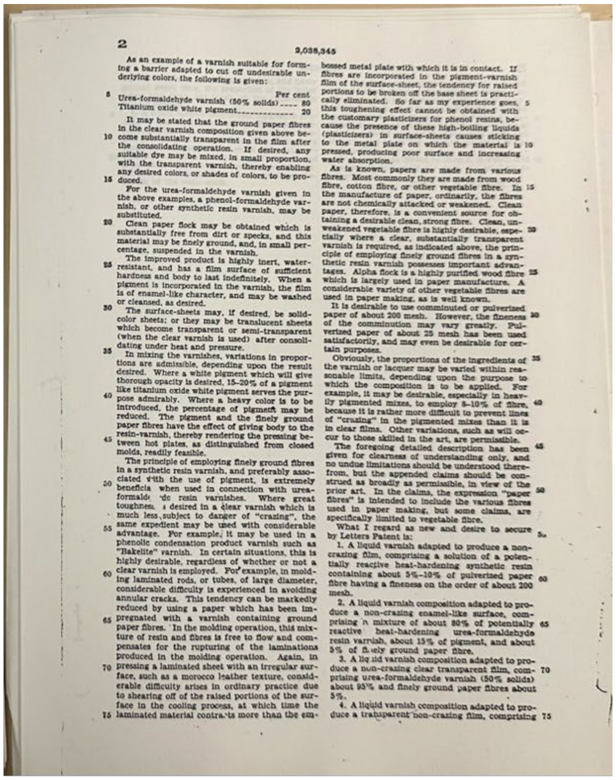

2

2,038,345

As an example of a varnish suitable for forming a barrier adapted to cut off undesirable underlying colors, the following is given:

	Per cent
Urea-formaldehyde varnish (50% solids)	80
Titanium oxide white pigment	20

It may be stated that the ground paper fibres in the clear varnish composition given above become substantially transparent in the film after the consolidating operation. If desired, any suitable dye may be mixed, in small proportion, with the transparent varnish, thereby enabling any desired colors, or shades of colors, to be produced.

For the urea-formaldehyde varnish given in the above examples, a phenol-formaldehyde varnish, or other synthetic resin varnish, may be substituted.

Clean paper flock may be obtained which is substantially free from dirt or specks, and this material may be finely ground, and, in small percentage, suspended in the varnish.

The improved product is highly inert, water-resistant, and has a film surface of sufficient hardness and body to last indefinitely. When a pigment is incorporated in the varnish, the film is of enamel-like character, and may be washed or cleansed, as desired.

The surface-sheets may, if desired, be solid-color sheets; or they may be translucent sheets which become transparent or semi-transparent (when the clear varnish is used) after consolidating under heat and pressure.

In mixing the varnishes, variations in proportions are admissible, depending upon the result desired. Where a white pigment which will give thorough opacity is desired, 15–20% of a pigment like titanium oxide white pigment serves the purpose admirably. Where a heavy color is to be introduced, the percentage of pigment may be reduced. The pigment and the finely ground paper fibres have the effect of giving body to the resin-varnish, thereby rendering the pressing between hot plates, as distinguished from closed molds, readily feasible.

The principle of employing finely ground fibres in a synthetic resin varnish, and preferably associated with the use of pigment, is extremely beneficia[illegible] when used in connection with urea-formald[illegible]de resin varnishes. Where great toughnes[illegible] is desired in a clear varnish which is much less subject to danger of "crazing", the same expedient may be used with considerable advantage. For example, it may be used in a phenolic condensation product varnish such as "Bakelite" varnish. In certain situations, this is highly desirable, regardless of whether or not a clear varnish is employed. For example, in molding laminated rods, or tubes, of large diameter, considerable difficulty is experienced in avoiding annular cracks. This tendency can be markedly reduced by using a paper which has been impregnated with a varnish containing ground paper fibres. In the molding operation, this mixture of resin and fibres is free to flow and compensates for the rupturing of the laminations produced in the molding operation. Again, in pressing a laminated sheet with an irregular surface, such as a morocco leather texture, considerable difficulty arises in ordinary practice due to shearing off of the raised portions of the surface in the cooling process, at which time the laminated material contracts more than the embossed metal plate with which it is in contact. If fibres are incorporated in the pigment-varnish film of the surface-sheet, the tendency for raised portions to be broken off the base sheet is practically eliminated. So far as my experience goes, this toughening effect cannot be obtained with the customary plasticizers for phenol resins, because the presence of these high-boiling liquids (plasticizers) in surface-sheets causes sticking to the metal plate on which the material is pressed, producing poor surface and increasing water absorption.

As is known, papers are made from various fibres. Most commonly they are made from wood fibre, cotton fibre, or other vegetable fibre. In the manufacture of paper, ordinarily, the fibres are not chemically attacked or weakened. Clean paper, therefore, is a convenient source for obtaining a desirable clean, strong fibre. Clean, unweakened vegetable fibre is highly desirable, especially where a clear, substantially transparent varnish is required, as indicated above, the principle of employing finely ground fibres in a synthetic resin varnish possesses important advantages. Alpha flock is a highly purified wood fibre which is largely used in paper manufacture. A considerable variety of other vegetable fibres are used in paper making, as is well known.

It is desirable to use comminuted or pulverized paper of about 200 mesh. However, the fineness of the comminution may vary greatly. Pulverized paper of about 25 mesh has been used satisfactorily, and may even be desirable for certain purposes.

Obviously, the proportions of the ingredients of the varnish or lacquer may be varied within reasonable limits, depending upon the purpose to which the composition is to be applied. For example, it may be desirable, especially in heavily pigmented mixes, to employ 8–10% of fibre, because it is rather more difficult to prevent lines of "crazing" in the pigmented mixes than it is in clear films. Other variations, such as will occur to those skilled in the art, are permissible.

The foregoing detailed description has been given for clearness of understanding only, and no undue limitations should be understood therefrom, but the appended claims should be construed as broadly as permissible, in view of the prior art. In the claims, the expression "paper fibres" is intended to include the various fibres used in paper making, but some claims, are specifically limited to vegetable fibre.

What I regard as new and desire to secure by Letters Patent is:

1. A liquid varnish adapted to produce a non-crazing film, comprising a solution of a potentially reactive heat-hardening synthetic resin containing about 5%–10% of pulverized paper fibre having a fineness on the order of about 200 mesh.

2. A liquid varnish composition adapted to produce a non-crazing enamel-like surface, comprising a mixture of about 80% of potentially reactive heat-hardening urea-formaldehyde resin varnish, about 15% of pigment, and about 5% of finely ground paper fibre.

3. A liquid varnish composition adapted to produce a non-crazing clear transparent film, comprising urea-formaldehyde varnish (50% solids) about 95% and finely ground paper fibres about 5%.

4. A liquid varnish composition adapted to produce a transparent non-crazing film, comprising

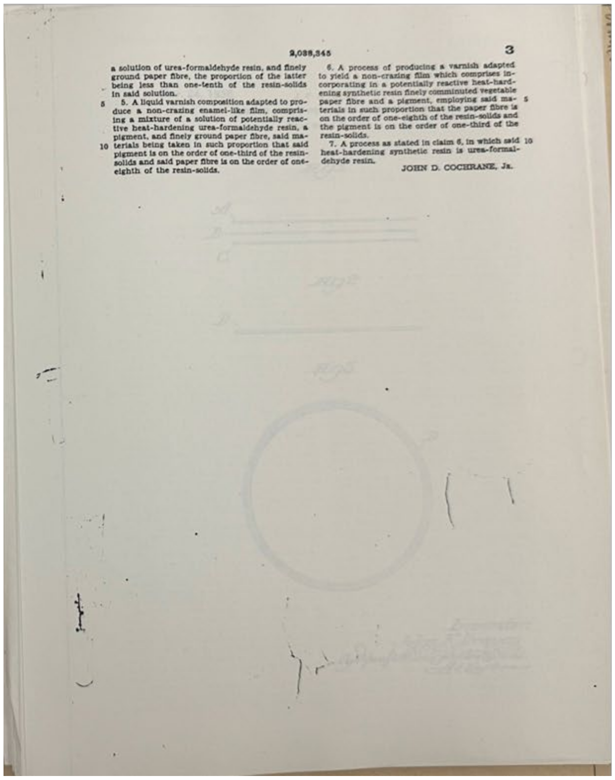

2,038,345

3

a solution of urea-formaldehyde resin, and finely ground paper fibre, the proportion of the latter being less than one-tenth of the resin-solids in said solution.

5. A liquid varnish composition adapted to produce a non-crazing enamel-like film, comprising a mixture of a solution of potentially reactive heat-hardening urea-formaldehyde resin, a pigment, and finely ground paper fibre, said materials being taken in such proportion that said pigment is on the order of one-third of the resin-solids and said paper fibre is on the order of one-eighth of the resin-solids.

6. A process of producing a varnish adapted to yield a non-crazing film which comprises incorporating in a potentially reactive heat-hardening synthetic resin finely comminuted vegetable paper fibre and a pigment, employing said materials in such proportion that the paper fibre is on the order of one-eighth of the resin-solids and the pigment is on the order of one-third of the resin-solids.

7. A process as stated in claim 6, in which said heat-hardening synthetic resin is urea-formaldehyde resin.

JOHN D. COCHRANE, Jr.

Figure S2: Patent No. 2,038,345

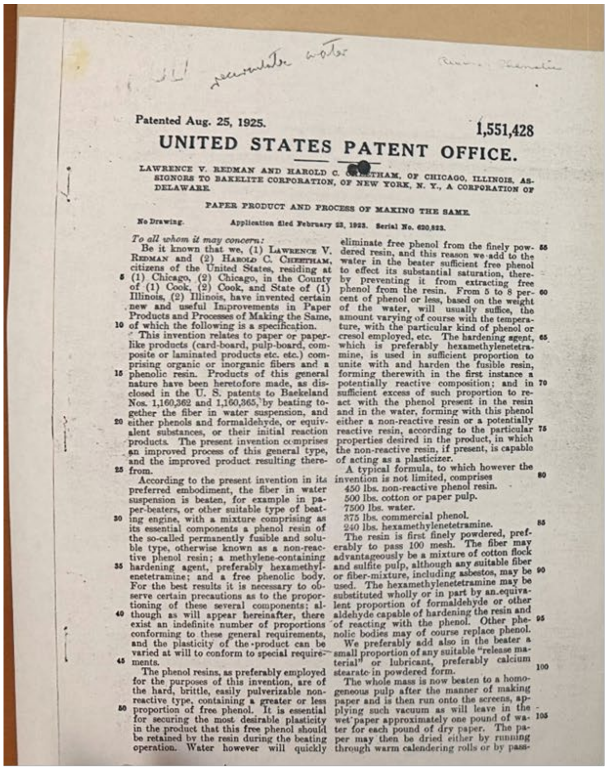

Patented Aug. 25, 1925. 1,551,428

UNITED STATES PATENT OFFICE.

LAWRENCE V. REDMAN AND HAROLD C. [illegible]THAM, OF CHICAGO, ILLINOIS, ASSIGNORS TO BAKELITE CORPORATION, OF NEW YORK, N. Y., A CORPORATION OF DELAWARE.

PAPER PRODUCT AND PROCESS OF MAKING THE SAME.

No Drawing. Application filed February 23, 1923. Serial No. 620,823.

To all whom it may concern:

Be it known that we, (1) LAWRENCE V. REDMAN and (2) HAROLD C. CHEETHAM, citizens of the United States, residing at (1) Chicago, (2) Chicago, in the County of (1) Cook, (2) Cook, and State of (1) Illinois, (2) Illinois, have invented certain new and useful Improvements in Paper Products and Processes of Making the Same, of which the following is a specification.

This invention relates to paper or paper-like products (card-board, pulp-board, composite or laminated products etc. etc.) comprising organic or inorganic fibers and a phenolic resin. Products of this general nature have been heretofore made, as disclosed in the U. S. patents to Baekeland Nos. 1,160,362 and 1,160,365, by beating together the fiber in water suspension, and either phenols and formaldehyde, or equivalent substances, or their initial reaction products. The present invention comprises an improved process of this general type, and the improved product resulting therefrom.

According to the present invention in its preferred embodiment, the fiber in water suspension is beaten, for example in paper-beaters, or other suitable type of beating engine, with a mixture comprising as its essential components a phenol resin of the so-called permanently fusible and soluble type, otherwise known as a non-reactive phenol resin; a methylene-containing hardening agent, preferably hexamethylenetetramine; and a free phenolic body. For the best results it is necessary to observe certain precautions as to the proportioning of these several components; although as will appear hereinafter, there exist an indefinite number of proportions conforming to these general requirements, and the plasticity of the product can be varied at will to conform to special requirements.

The phenol resins, as preferably employed for the purposes of this invention, are of the hard, brittle, easily pulverizable non-reactive type, containing a greater or less proportion of free phenol. It is essential for securing the most desirable plasticity in the product that this free phenol should be retained by the resin during the beating operation. Water however will quickly eliminate free phenol from the finely powdered resin, and this reason we add to the water in the beater sufficient free phenol to effect its substantial saturation, thereby preventing it from extracting free phenol from the resin. From 5 to 8 per cent of phenol or less, based on the weight of the water, will usually suffice, the amount varying of course with the temperature, with the particular kind of phenol or cresol employed, etc. The hardening agent, which is preferably hexamethylenetetramine, is used in sufficient proportion to unite with and harden the fusible resin, forming therewith in the first instance a potentially reactive composition; and in sufficient excess of such proportion to react with the phenol present in the resin and in the water, forming with this phenol either a non-reactive resin or a potentially reactive resin, according to the particular properties desired in the product, in which the non-reactive resin, if present, is capable of acting as a plasticizer.

A typical formula, to which however the invention is not limited, comprises

450 lbs. non-reactive phenol resin.
500 lbs. cotton or paper pulp.
7500 lbs. water.
375 lbs. commercial phenol.
240 lbs. hexamethylenetetramine.

The resin is first finely powdered, preferably to pass 100 mesh. The fiber may advantageously be a mixture of cotton flock and sulfite pulp, although any suitable fiber or fiber-mixture, including asbestos, may be used. The hexamethylenetetramine may be substituted wholly or in part by an equivalent proportion of formaldehyde or other aldehyde capable of hardening the resin and of reacting with the phenol. Other phenolic bodies may of course replace phenol.

We preferably add also in the beater a small proportion of any suitable "release material" or lubricant, preferably calcium stearate in powdered form.

The whole mass is now beaten to a homogeneous pulp after the manner of making paper and is then run onto the screens, applying such vacuum as will leave in the wet paper approximately one pound of water for each pound of dry paper. The paper may then be dried either by running through warm calendering rolls or by pass-

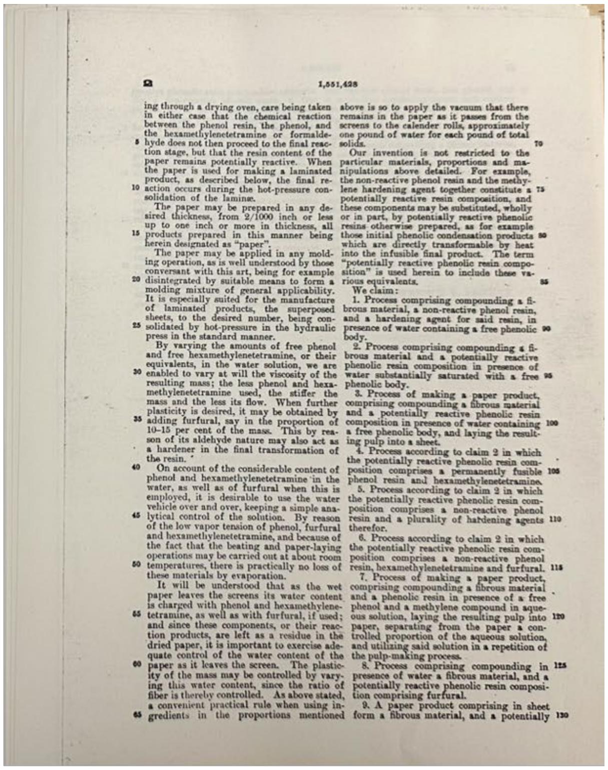

2 1,551,428

ing through a drying oven, care being taken in either case that the chemical reaction between the phenol resin, the phenol, and the hexamethylenetetramine or formaldehyde does not then proceed to the final reaction stage, but that the resin content of the paper remains potentially reactive. When the paper is used for making a laminated product, as described below, the final reaction occurs during the hot-pressure consolidation of the laminæ.

The paper may be prepared in any desired thickness, from 2/1000 inch or less up to one inch or more in thickness, all products prepared in this manner being herein designated as "paper".

The paper may be applied in any molding operation, as is well understood by those conversant with this art, being for example disintegrated by suitable means to form a molding mixture of general applicability. It is especially suited for the manufacture of laminated products, the superposed sheets, to the desired number, being consolidated by hot-pressure in the hydraulic press in the standard manner.

By varying the amounts of free phenol and free hexamethylenetetramine, or their equivalents, in the water solution, we are enabled to vary at will the viscosity of the resulting mass; the less phenol and hexamethylenetetramine used, the stiffer the mass and the less its flow. When further plasticity is desired, it may be obtained by adding furfural, say in the proportion of 10–15 per cent of the mass. This by reason of its aldehyde nature may also act as a hardener in the final transformation of the resin.

On account of the considerable content of phenol and hexamethylenetetramine in the water, as well as of furfural when this is employed, it is desirable to use the water vehicle over and over, keeping a simple analytical control of the solution. By reason of the low vapor tension of phenol, furfural and hexamethylenetetramine, and because of the fact that the beating and paper-laying operations may be carried out at about room temperatures, there is practically no loss of these materials by evaporation.

It will be understood that as the wet paper leaves the screens its water content is charged with phenol and hexamethylenetetramine, as well as with furfural, if used; and since these components, or their reaction products, are left as a residue in the dried paper, it is important to exercise adequate control of the water content of the paper as it leaves the screen. The plasticity of the mass may be controlled by varying this water content, since the ratio of fiber is thereby controlled. As above stated, a convenient practical rule when using ingredients in the proportions mentioned above is so to apply the vacuum that there remains in the paper as it passes from the screens to the calender rolls, approximately one pound of water for each pound of total solids.

Our invention is not restricted to the particular materials, proportions and manipulations above detailed. For example, the non-reactive phenol resin and the methylene hardening agent together constitute a potentially reactive resin composition, and these components may be substituted, wholly or in part, by potentially reactive phenolic resins otherwise prepared, as for example those initial phenolic condensation products which are directly transformable by heat into the infusible final product. The term "potentially reactive phenolic resin composition" is used herein to include these various equivalents.

We claim:

1. Process comprising compounding a fibrous material, a non-reactive phenol resin, and a hardening agent for said resin, in presence of water containing a free phenolic body.

2. Process comprising compounding a fibrous material and a potentially reactive phenolic resin composition in presence of water substantially saturated with a free phenolic body.

3. Process of making a paper product, comprising compounding a fibrous material and a potentially reactive phenolic resin composition in presence of water containing a free phenolic body, and laying the resulting pulp into a sheet.

4. Process according to claim 2 in which the potentially reactive phenolic resin composition comprises a permanently fusible phenol resin and hexamethylenetetramine.

5. Process according to claim 2 in which the potentially reactive phenolic resin composition comprises a non-reactive phenol resin and a plurality of hardening agents therefor.

6. Process according to claim 2 in which the potentially reactive phenolic resin composition comprises a non-reactive phenol resin, hexamethylenetetramine and furfural.

7. Process of making a paper product, comprising compounding a fibrous material and a phenolic resin in presence of a free phenol and a methylene compound in aqueous solution, laying the resulting pulp into paper, separating from the paper a controlled proportion of the aqueous solution, and utilizing said solution in a repetition of the pulp-making process.

8. Process comprising compounding in presence of water a fibrous material, and a potentially reactive phenolic resin composition comprising furfural.

9. A paper product comprising in sheet form a fibrous material, and a potentially

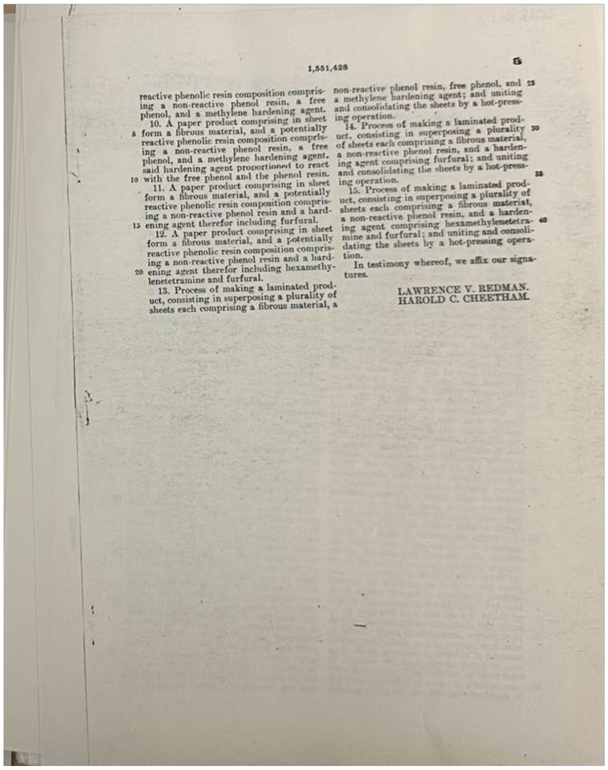

1,551,428 3

reactive phenolic resin composition comprising a non-reactive phenol resin, a free phenol, and a methylene hardening agent.

10. A paper product comprising in sheet form a fibrous material, and a potentially reactive phenolic resin composition comprising a non-reactive phenol resin, a free phenol, and a methylene hardening agent, said hardening agent proportioned to react with the free phenol and the phenol resin.

11. A paper product comprising in sheet form a fibrous material, and a potentially reactive phenolic resin composition comprising a non-reactive phenol resin and a hardening agent therefor including furfural.

12. A paper product comprising in sheet form a fibrous material, and a potentially reactive phenolic resin composition comprising a non-reactive phenol resin and a hardening agent therefor including hexamethylenetetramine and furfural.

13. Process of making a laminated product, consisting in superposing a plurality of sheets each comprising a fibrous material, a non-reactive phenol resin, free phenol, and a methylene hardening agent; and uniting and consolidating the sheets by a hot-pressing operation.

14. Process of making a laminated product, consisting in superposing a plurality of sheets each comprising a fibrous material, a non-reactive phenol resin, and a hardening agent comprising furfural; and uniting and consolidating the sheets by a hot-pressing operation.

15. Process of making a laminated product, consisting in superposing a plurality of sheets each comprising a fibrous material, a non-reactive phenol resin, and a hardening agent comprising hexamethylenetetramine and furfural; and uniting and consolidating the sheets by a hot-pressing operation.

In testimony whereof, we affix our signatures.

LAWRENCE V. REDMAN.
HAROLD C. CHEETHAM.

Figure S3: Patent No. 1,551,428

All images of the selected patent were obtained from research on the Formica Collection, Arch. Cent. Natl. Mus. Am. Hist. (n.d.).

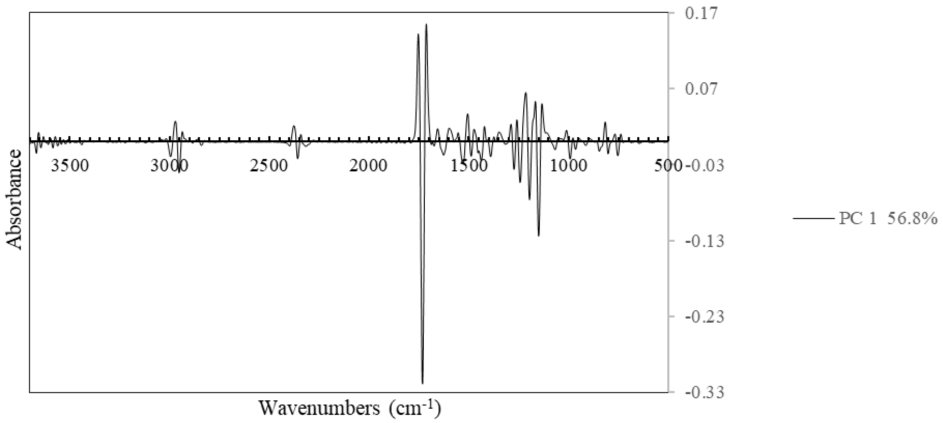

Figure S4: Plot of loading for Principal Component 1

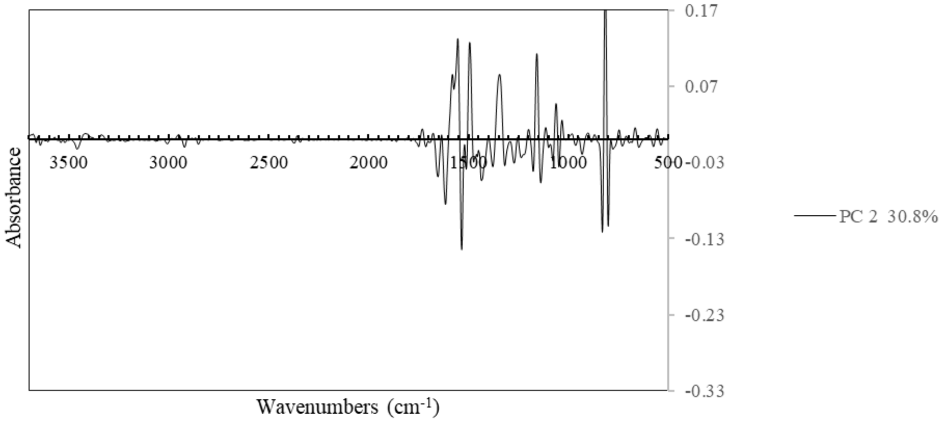

Figure S5: Plot of loading for Principal Component 2

	Explained variance %	*eigenvalues*
PC 1	56.75353	0.003321
PC 2	30.78191	0.001801
PC 3	5.286529	0.000309
PC 4	2.540725	0.000149
PC 5	1.61642	9.46E-05
PC 6	1.046669	6.12E-05
PC 7	0.708811	4.15E-05
PC 8	0.615435	0.000036
PC 9	0.380561	2.23E-05
PC10	0.269412	1.58E-05

Table S1: Explained variance percentage and eigenvalues for all 10 principal components
